# Genomic analysis reveals multi-level resistance network linking oxidative stress pathway mutations to cefiderocol resistance and clonal evolution in *Klebsiella pneumoniae* from the United Arab Emirates

**DOI:** 10.3389/fcimb.2026.1820198

**Published:** 2026-05-15

**Authors:** Lana Daoud, Timothy Collyns, Akela Ghazawi, Farah Al-Marzooq

**Affiliations:** 1 Department of Medical Microbiology and Immunology, College of Medicine and Health Sciences, United Arab Emirates University, Al-Ain, United Arab Emirates; 2 Tawam Hospital, Al-Ain, United Arab Emirates

**Keywords:** antimicrobial resistance, cefiderocol, iron transport, *Klebsiella pneumoniae*, oxidative stress, UAE, whole-genome sequencing

## Abstract

**Introduction:**

Resistance to cefiderocol (CFDC), a novel siderophore-cephalosporin, remains poorly understood. We aimed to explore mutations in oxidative stress (OS) pathways in *Klebsiella pneumoniae* (KPN) and investigate their potential association with reduced susceptibility to CFDC.

**Methods:**

A total of 282 KPN strains from the United Arab Emirates were tested for susceptibility to various antibiotics, including CFDC. Afterward, 96 representative strains were subjected to whole-genome sequencing and bioinformatic analyses to examine 51 genes across six functional categories (OxyR, SoxRS, iron regulation, efflux pumps, DNA repair, and penicillin-binding proteins). Genotypic and phenotypic results were correlated to explore significant associations with CFDC susceptibility.

**Results:**

CFDC demonstrated high *in vitro* activity (96.9%), with eight isolates showing non-susceptibility. All the CFDC-resistant isolates carried *bla*_NDM_ carbapenemases and belonged to high-risk clones (primarily ST14 and ST147), with enrichment of OS pathway mutations. Mutations in iron uptake genes, especially *cirA* frameshift insertion, were enriched in resistant isolates. Mutations in OS detoxification (mainly the OxyR regulon), efflux pump, and DNA repair pathways further contributed to altered susceptibility profiles. The negative correlations between the MICs of CFDC and mutations in detoxification and repair genes suggested that impaired stress responses increase CFDC activity. Network analysis revealed that resistance emerged through coordinated multi-pathway interactions, with iron regulation genes serving as the central hub. High-risk clones exhibited dense co-mutation networks spanning genes involved in iron uptake, OS response, and efflux systems, indicating a multifactorial adaptive strategy.

**Conclusions:**

Coordinated interactions across iron regulation, OS, and efflux pathways are associated with reduced susceptibility to CFDC. The negative correlation between OxyR/DNA repair mutations and CFDC MICs suggests that intact OS defenses paradoxically reduce CFDC efficacy. OS pathway integrity may modulate CFDC susceptibility, informing future strategies to preserve CFDC efficacy. The identification of specific mutations linked to CFDC resistance provides potential biomarkers for surveillance and therapeutic targeting, underscoring the interplay between OS responses and antimicrobial resistance.

## Introduction

*Klebsiella pneumoniae* (KPN) is an opportunistic pathogen that causes a wide spectrum of community- and hospital-acquired infections, such as pneumonia, urinary tract infections, and bloodstream infections (Ashurst and Dawson, 2021). Its pathogenicity is compounded by its increasing resistance to multiple antibiotic classes, rendering many treatment options ineffective (Pitout et al., 2015). KPN is among the pathogens most likely to cause antimicrobial resistance (AMR)-related mortality over the past few decades (Shah et al., 2025). KPN is intrinsically resistant to ampicillin due to the presence of chromosomally encoded *bla*_SHV_ variants, which are nearly universally present in the species and confer a baseline level of β-lactam resistance. It typically harbors several acquired AMR genes that encode degradative enzymes that hydrolyze a broad spectrum of β-lactam antibiotics, such as extended-spectrum β-lactamases (ESBLs), including *bla*_CTX-M_, which confer resistance to cephalosporins, and carbapenemases such as *bla*_OXA-48_*_-like_*, *bla*_KPC_, *bla*_NDM_, *bla*_VIM_, and *bla*_IMP_, which confer resistance to carbapenems (Poole, 2012; Pitout et al., 2015). Other causes of resistance include chromosomal mutations that affect membrane permeability and efflux pump systems, thereby decreasing intracellular antibiotic concentrations.

Intrinsic bacterial stress response systems can also contribute to AMR phenotypes (Poole, 2012; Dam et al., 2018). One such system is the oxidative stress (OS) response, which is a critical survival mechanism activated when bacteria encounter reactive oxygen species (ROS). ROS are reactive, unstable oxygen molecules that can react with cellular macromolecules, such as DNA, resulting in extensive damage to critical cellular components and ultimately leading to cell death (Borisov et al., 2021). ROS can arise from exogenous sources, such as bactericidal antibiotics, environmental stress, and immune cell attack (Kohanski et al., 2007; Dwyer et al., 2014; Canton et al., 2021). Antibiotics such as β-lactams, fluoroquinolones, and aminoglycosides are known to indirectly increase intracellular ROS levels through metabolic disruption and redox imbalance (Sha and Schacht, 1999; Masadeh et al., 2017; Léger et al., 2019). KPN, like other bacteria, has several defense mechanisms to survive ROS toxicity, which rely on the robust activity of OS response systems. These systems not only allow persistence within an ROS-enriched phagolysosome but also may influence antibiotic susceptibility patterns through complex regulatory cross-talk and adaptive responses (Fang, 2004). OxyR, SoxRS, and MarRAB are three major regulons that orchestrate the OS response in KPN. OxyR, SoxR, and MarR are transcriptional regulators of these regulons and activate the expression of downstream detoxification genes upon induction.

Iron is involved in numerous processes in bacterial cells and hence is crucial for their survival and metabolism. It is also notorious as an ROS inducer, as it catalyzes the generation of hydroxyl radicals from hydrogen peroxide (H_2_O_2_) in Fenton chemistry (Jones, 2007). This toxic mechanism can enhance the activity of antimicrobials, aiding in the eradication of infections (Abeydeera et al., 2023). Iron homeostasis is crucial for bacteria to avoid the toxic effects of extracellular iron and, consequently, prevent the occurrence of the Fenton reaction. In KPN, the *fur* gene is a central component of the FUR regulon (Troxell and Hassan, 2013). During iron abundance, Fur forms a complex with Fe^2+^ and represses the transcription of genes involved in iron entry. During iron starvation, this complex dissociates and detaches from the promoter region to allow the transcription of iron acquisition genes (Cornelis et al., 2011). These genes play important roles in the activity of a novel siderophore-conjugated cephalosporin, cefiderocol (CFDC), as they assist in the uptake of the drug through the outer membrane (Ito et al., 2016; Daoud et al., 2022).

CFDC shows promising therapeutic activity against multidrug-resistant (MDR) Gram-negative bacteria (GNB), including KPN (Daoud et al., 2023; Falagas et al., 2017). The efficacy of CFDC has been well documented in recent years, demonstrating its superiority over other potent antibiotics used to treat GNB infections. It has the ability to chelate iron in the environment, creating a complex that can be actively transported into cells via iron transport receptors, thereby overcoming conventional mechanisms of resistance, such as outer membrane mutations and efflux pumps (Daoud et al., 2022; Repac Antić et al., 2022). Recent reports have shown that treatment with CFDC chelates extracellular ferric iron via its catechol moiety, which is then actively transported into bacterial cells via TonB-dependent iron receptors. CFDC utilizes siderophore-mediated iron transport systems for cellular uptake (Ito et al., 2016), and ferric iron transport pathways influence susceptibility (Polani et al., 2025). A recent study in *Escherichia coli* suggested that CFDC treatment can increase intracellular iron and ROS levels, based on proteomic and biochemical analyses (Du et al., 2022). These findings support the possibility that iron-mediated oxidative damage may contribute to CFDC bactericidal activity, although this mechanism requires further validation. Nevertheless, this potential mechanism of action has not been thoroughly explored in the context of clinical resistance in KPN, where existing studies have mostly focused on enzymatic β-lactamase-mediated drug degradation and defects in iron transport genes such as *cirA*. While OS pathways have been linked to resistance against other antibiotic classes, including β-lactams, fluoroquinolones, and aminoglycosides (Poole, 2012; Dwyer et al., 2014; Brand et al., 2025), their role in modulating CFDC susceptibility remains poorly investigated. We therefore hypothesize that intracellularly introduced iron by CFDC drives ROS generation via the Fenton reaction, and that OS pathway mutations represent an underexplored yet mechanistically important determinant of CFDC susceptibility. The potential intersection between OS response systems and AMR is increasingly being recognized, and the evidence from studies on enterobacteria suggests that mutations in OS-related genes can modulate antibiotic susceptibility. To support this hypothesis, this study was conducted with the aim of investigating the association between mutations in OS response genes and AMR phenotypes in clinical isolates of KPN collected over multiple years from a tertiary hospital in the United Arab Emirates (UAE). Furthermore, we aimed to investigate the association of these mutations with altered CFDC susceptibility, along with other genetic factors contributing to CFDC resistance, including iron transport genes and β-lactamase resistance mechanisms.

## Materials and methods

### Bacterial isolates

A total of 282 KPN strains were collected from patients attending Tawam Hospital in Al-Ain, UAE,between 2021 and 2024. These strains were aseptically isolated from urine (n=115; 40.8%), respiratory samples (n=65; 23%), blood (n=30; 10.6%), wounds (n=30; 10.6%), body fluid (n=14; 5%), skin (n=9; 3.2%), rectal swabs (n=9; 3.2%), tissue (n=6; 2.1%), and other types of samples (n= 4; 1.4%). Detailed strain information is provided in **Supplementary Data File 1**. Clinical isolates were stored in brain heart infusion broth supplemented with 20% glycerol at −80 °C for long-term preservation (Al-Marzooq et al., 2025b).

### Antimicrobial susceptibility testing

Minimum inhibitory concentrations (MICs) were determined using the microbroth dilution assay and were interpreted according to the Clinical and Laboratory Standards Institute (CLSI) breakpoints (CLSI, 2026). Thirteen antibiotics representing 9 classes used to treat GNB were tested. These include polymyxin E [colistin (COL)], aminoglycosides [gentamicin (GM), amikacin (AK)], tetracyclines [tetracycline (TET), tigecycline (TGC)], fluoroquinolones [ciprofloxacin (CIP)], cephalosporins [ceftazidime (CAZ), cefepime (CPM), cefotaxime (CTX)], a β-lactam/β-lactamase inhibitor combination [ceftazidime/avibactam (CAZ/AVI)], a carbapenem [meropenem (MEM)], a monobactam [aztreonam (AZT)], and CFDC. CFDC was tested using iron-depleted, cation-adjusted Mueller-Hinton broth (Daoud et al., 2023). The iron concentration in the medium was precisely determined to be 0.36 nM using an iron assay kit (cat. no. ab83366; Abcam, UK). The isolates were grouped on the basis of their overall drug resistance profiles following the definition of Magiorakos et al. (2012), i.e., resistance to at least one drug in three antibiotic classes was denoted as multidrug-resistant (MDR), and when resistant to more than six antibiotic classes, the strains were considered to be extensively drug resistant (XDR) (Magiorakos et al., 2012); otherwise, the strains were considered susceptible (S). Moreover, the strains were grouped on the basis of their CFDC susceptibility profiles as follows: CFDC-susceptible (CFDC-S), CFDC-intermediately susceptible (CFDC-I), and CFDC-resistant (CFDC-R).

### Whole-genome sequencing and bioinformatics analysis

A total of 96 strains were selected for WGS to represent a broad range of resistance characteristics. All CFDC non-susceptible isolates (n=8) were included. Within the XDR group, isolates were selected to include varied carbapenem resistance profiles, including both carbapenem-resistant, non-carbapenem-resistant KPN and dual carbapenemase producers. Within the MDR group, isolates were selected to represent a range of CFDC MICs. A subset of susceptible isolates was also included to enable comparison of mutation profiles between resistant and susceptible strains. This selection resulted in a final subset of 16 S, 27 MDR, and 53 XDR isolates, enriched for XDR to facilitate the analysis of resistance mechanisms.

DNA was extracted from overnight bacterial cultures grown in Luria-Bertani (LB) broth. A volume of 1 ml of the bacterial suspension (~2×10^9^ CFU/ml) was centrifuged to pellet the cells for extraction using the Wizard^®^ Genomic DNA Purification Kit (Promega, USA) following the manufacturer’s instructions. WGS was performed using short-read sequencing on the DNBSEQ-G400RS platform (MGI-Tech, Hong Kong). Genome assemblies were generated using Unicycler version 0.5.0. (https://github.com/rrwick/Unicycler). Multilocus sequence typing (MLST) was performed *in silico* using assembled genome sequences to determine the sequence types (STs) of the isolates. The resulting MLST data were subsequently used to assess clonal diversity. To assess the clonality and phylogenetic relationships of CFDC non-susceptible isolates, whole-genome single-nucleotide polymorphism (SNP)-based phylogenetic analysis was performed. Raw sequencing reads for all 96 isolates were mapped against the reference genome MGH78578 using Snippy v4.0.2 (https://github.com/tseemann/snippy). SNP calling was conducted with default parameters, and consensus sequences were generated for each isolate. Core genome SNP sites present across all isolates were extracted and aligned using snp-sites v2.5.1. Pairwise SNP distances between all isolates were calculated using snp-dists v0.8.2. A maximum-likelihood phylogenetic tree was constructed from the core SNP alignment using IQ-TREE 3.0.1 with the GTR+F+G4 nucleotide substitution model. Branch support was assessed using ultrafast bootstrap approximation with 1,000 replicates. The tree was visualized and annotated using iTOL v6/ggtree v3.16.0. Clonality was assessed based on pairwise SNP distances, with isolates differing by ≤20 SNPs considered potentially clonally related, consistent with thresholds applied in previous genomic epidemiology studies of KPN (Bowers et al., 2015; Rodrigues et al., 2023; Feng et al., 2024).

Antimicrobial resistance genes were identified using the ResFinder tool hosted by the Center for Genomic Epidemiology (https://github.com/genomicepidemiology/resfinder). Assembled sequences were annotated using the Rapid Annotations using Subsystems Technology (RAST) server version 1.3.0 and Prokka v1.14.6 (https://github.com/tseemann/prokka) (Aziz et al., 2008; Seemann, 2014). DNA sequences were translated into amino acid sequences (AA) and aligned against a reference KPN strain, MGH 78578 (ATCC 700721), to identify mutations across the annotated genes of interest. Outer membrane porin (OMPs) genes (OmpK35 and OmpK36) were analyzed using Kleborate v2.3.2 to identify disruptions in the genes (Lam et al., 2021).

### Identification of oxidative stress-related genes

**Figure 1:**
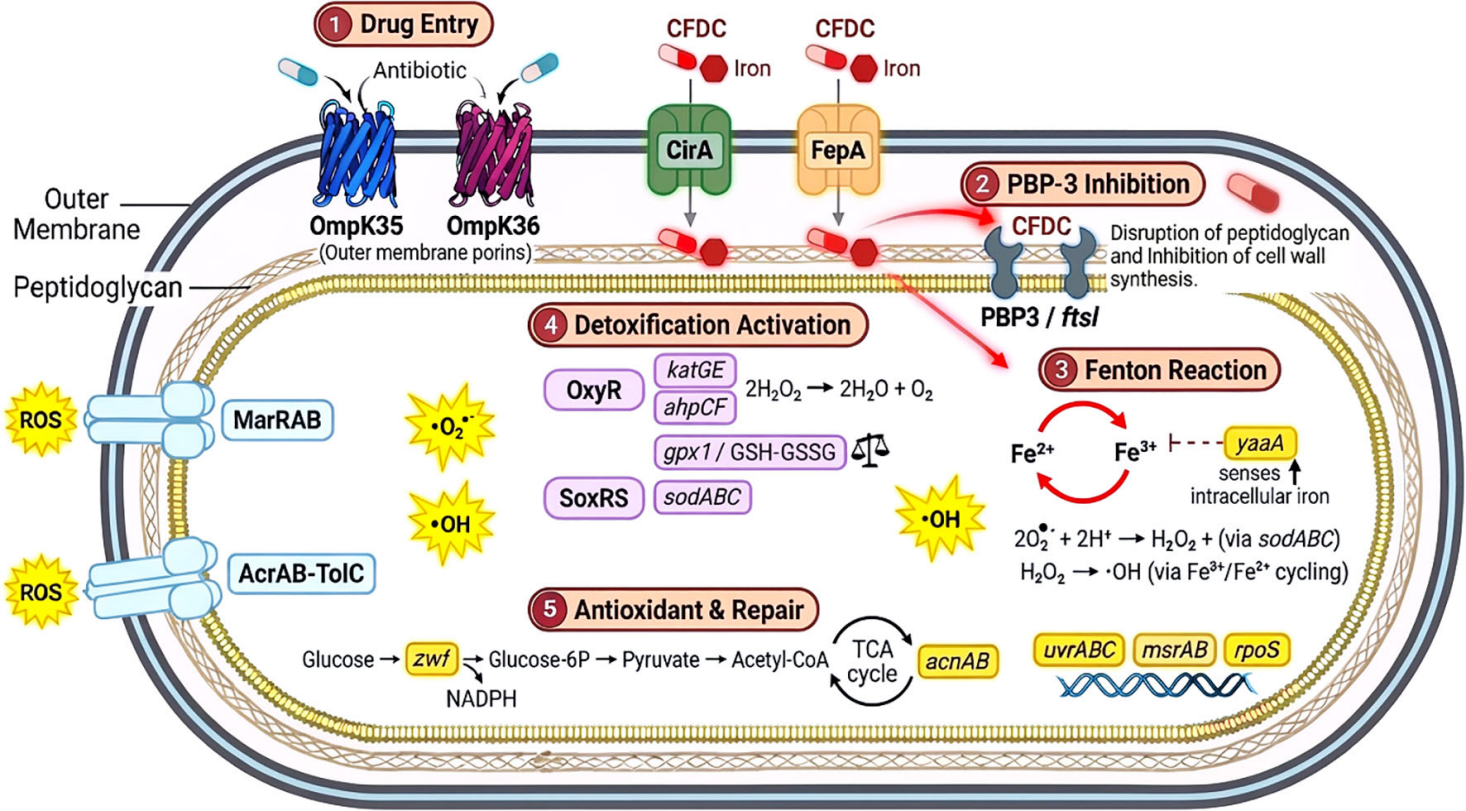
Hypothesized mechanistic model of CFDC antibacterial activity in *K. pneumoniae*. Step 1: CFDC chelates extracellular ferric iron via its catechol moiety and gains entry into the periplasmic space through TonB-dependent iron uptake receptors (CirA, FepA) and passively through outer membrane porins (OmpK35/OmpK36). Step 2: once in the periplasm, CFDC binds penicillin-binding protein 3 (PBP3- *ftsI*) and inhibits cell wall production. Step 3: iron released from the CFDC-iron complex in the periplasm is hypothesized to enter the cytoplasm, where it catalyzes the Fenton reaction, converting H_2_O_2_ into highly toxic hydroxyl radicals (•OH). The *yaaA* gene product senses the elevation in intracellular iron and acts to limit further ROS generation. Step 4: accumulating ROS activates detoxification systems, OxyR upregulates *katG, katE*, and *ahpCF* to detoxify H_2_O_2_, and *gpx1*/glutathione (GSH/GSSG) for redox balance, while SoxRS induces *sodABC* (converting O_2_•^-^ to H_2_O_2_) and efflux pumps (*marRAB*) to expel toxic compounds. Step 5: antioxidant and repair responses are engaged, including NADPH generation via *zwf* (pentose phosphate pathway), TCA cycle activity (*acnAB*), and DNA damage repair (*uvrABC, msrAB, rpoS*) to restore redox homeostasis and genome integrity. Yellow symbols represent ROS species.

**Table 1** presents 51 OS-related genes that were selected on the basis of their involvement in cellular defenses, regulatory mechanisms, iron regulation, and cell wall synthesis (penicillin-binding protein, mainly PBP-3) and were categorized into six functional groups. These genes are depicted in **Figure 1**, which presents a proposed mechanistic model of CFDC antibacterial activity against *K. pneumoniae*.

**Table 1:** Genes investigated based on six functional pathways.

Category/regulon	Gene	Function
OxyR	*oxyR*	Hydrogen peroxide sensor and transcriptional activator
	*katE*	Catalase enzyme detoxifying H2O2 during stationary phase
	*katG*	Catalase-peroxidase involved in H2O2 detoxification
	*ahpF*	Provides reducing equivalents to AhpC for peroxide detoxification
	*ahpC*	Reduces organic peroxides via alkyl hydroperoxide reductase system
	*grxA*	Cytoplasmic glutaredoxin for redox balance
	*trxC*	Thioredoxin involved in disulfide bond reduction
	*trxC2*	Putative thioredoxin isoform; redox homeostasis
	*gpx1*	Putative glutathione peroxidase for organic peroxide detoxification
	*yaaA*	Reduces free intracellular iron and suppress Fenton reaction
SoxRS	*soxR*	Redox-sensitive transcriptional regulator of superoxide response
	*soxS*	Activates SoxRS-dependent antioxidant response genes
	*sodA*	Cytoplasmic superoxide dismutase detoxifying O_2_^-^ radicals
	*sodB*	Fe-superoxide dismutase involved in ROS detoxification
	*sodC*	Periplasmic Cu/Zn superoxide dismutase
	*acnA*	TCA cycle enzyme sensitive to oxidative stress and iron-sulfur damage
	*acnB*	Aconitase B isoform, involved in the TCA cycle and redox regulation
	*zwf*	Glucose-6-phosphate dehydrogenase; supplies NADPH for ROS detox
	*btuE*	Glutathione peroxidase protecting against lipid peroxides
	*grxD*	Putative glutaredoxin involved in oxidative stress protection
	*grxC*	Glutaredoxin-like protein involved in redox regulation
	*trxA*	Thioredoxin that reduces disulfide bonds in cytoplasmic proteins
	*trxA2*	Thioredoxin isoform involved in oxidative stress response
	*trxA3*	Additional thioredoxin isoform with similar function
	*trxB*	Thioredoxin reductase restoring reduced thioredoxin pool
Iron regulation	*fur*	Ferric uptake regulator; represses iron acquisition systems
	*fpr*	Ferredoxin-NADPH reductase generating NADPH for oxidative defense
	*cirA*	TonB-dependent transporter for siderophores
	*fief*	Cation efflux pump for iron and zinc detoxification
	*fiu*	Siderophore receptor for iron acquisition
	*fepA_1*	Ferric enterobactin outer membrane receptor
	*fepA_2*	Alternative ferric enterobactin receptor isoform
	*sitA*	Iron/manganese ABC transporter substrate-binding protein
	*feoA*	Ferrous iron transport system component
	*feoB*	GTPase driving ferrous iron uptake
	*fecA*	Ferric citrate outer membrane transporter
Efflux System	*tolC*	Outer membrane channel in multidrug efflux systems
	*marA*	Activator of multiple antibiotic resistance and efflux pumps
	*marB*	Modulates MarRAB regulon involved in multidrug resistance
	*marR*	Transcriptional regulator of the MarRAB regulon
	*acrA*	Membrane fusion protein in AcrAB-TolC efflux pump
	*acrB*	Multidrug transporter protein
	*kefC*	Potassium efflux system responding to toxic electrophiles
Repair	*msrA*	Repairs oxidized methionine (Met-SO) residues in proteins
	*msrB*	Reduces oxidized methionine (Met-RO) residues to functional form
	*rpoS*	Global stress response sigma factor regulating ROS genes
	*uvrA*	Initiates nucleotide excision repair of oxidatively damaged DNA
	*uvrB*	DNA helicase involved in excision repair complex
	*uvrC*	Endonuclease that cleaves damaged DNA strand
PBP-3	ftsI*a	Penicillin-binding protein 3- copy1
	ftsI*b	Penicillin-binding protein 3- copy2

The OxyR regulon comprises key components, including *oxyR*, *ahpC*, *ahpF*, *katG*, and *katE*, which play central roles in H_2_O_2_ sensing and detoxification. Additional redox regulators, such as *grxA*, *trxC*, *trxC2*, and *gpx1*, were also examined for their contributions to thiol-disulfide balance and peroxide defense. The SoxRS system was represented by genes like *soxR*, *soxS*, and *sodA*, as well as TCA-linked enzymes *acnA* and *acnB*, highlighting the intersection between OS response and metabolic regulation. Since CFDC acts as a siderophore, iron regulatory genes such as *fur*, *feoA*, *feoB*, *fiu*, and *fepA* variants were included to reflect the critical role of metal regulation in oxidative defense. Part of the detoxification process involves the elimination of ROS as well as antimicrobials via the efflux system, and hence *acrA*, *acrB*, *tolC*, *marA*, *marB*, and *kefC* were studied. DNA repair proteins encoded in *uvrA*, *uvrB*, *msrA*, and *msrB* were assessed for their potential contribution to stress resilience. Lastly, genes encoding PBP-3 (*ftsI*) were incorporated due to their role in maintaining cell wall integrity under stress conditions, and as they are the targets of CFDC. We identified 2 copies of the *ftsI* genes that were denoted as ftsI*a and ftsI*b.

### Statistical analyses

Statistical analysis of genotypic data was conducted in R software version 4.5.0 and Statistical Package for the Social Sciences version 29 (IBM, USA). A p value ≤0.05 was considered to indicate statistical significance. The chi-square test was employed to assess the association between the presence of mutations and resistance phenotypes. Standardized residual analysis was subsequently performed on significant chi-square results to measure the difference between observed and expected counts, scaled by the standard error, where values beyond ±2 indicate significant over- or underrepresentation, respectively (Sharpe, 2015). Additionally, the Kruskal–Wallis test was used to compare distributions across clinical groups (CFDC susceptibility, AMR phenotype, and STs). *Post hoc* pairwise comparisons were conducted when appropriate (Bhutani et al., 2016). Shannon diversity analysis was performed to evaluate the distribution of sequence types (STs) across AMR and CFDC susceptibility groups (Shannon, 1948). Spearman’s correlation analysis was also performed between gene mutation counts per group and MIC values, as well as AMR gene counts. In addition, pairwise correlation analysis between individual mutations was performed using Spearman’s rank method to generate a mutation co-occurrence network (Gao et al., 2022). A heatmap was constructed to illustrate the interactions between pathways, using the Phi coefficient (φ) as a measure of the strength of the association between two variables. It is the mean square contingency coefficient, which is calculated by dividing the chi-square value by the sample size and then taking the square root of the result. φ values >0.25 indicate a very strong correlation, >0.15 for strong correlations, >0.1 for moderate correlation, >0.05 for weak correlation, and >0 for no or very weak correlations (Akoglu, 2018). To explore the co-occurrence of mutations across the 6 pathways, we constructed a pathway-level correlation matrix based on pairwise mutation associations measured by the φ coefficient.

Furthermore, the association between individual mutations and changes in the CFDC MIC was examined using Wilcoxon rank-sum tests. Cohen’s d score was computed to estimate effect size, and the fold change in the MIC was calculated between the mutation-present and mutation-absent groups. Benjamini–Hochberg correction was applied to adjust for multiple testing, with an adjusted p-value (FDR) < 0.05 considered to indicate statistical significance (Benjamini and Hochberg, 1995).

To investigate the potential cooperative effects of co-occurring mutations on the MIC, we implemented a φ–coefficient–based strategy to quantify the link between the co-occurrence and MIC values. Pairs of mutations, each present in ≥3 isolates, were tested for association with increased MICs. MIC values in isolates with vs. without each mutation pair were compared. Statistical significance was assessed using chi-square tests (when all expected cell counts >5) or Fisher’s exact tests. Adjusted p-values were computed using the Benjamini–Hochberg method. Pairs with an adjusted p-value <0.05 and an absolute φ coefficient> 0.15 were considered potentially synergistic.

Data visualization was carried out using several R packages, including ggplot2 (version 3.5.2) for bar and violin plots, pheatmap (version 1.0.12) for heatmap generation, circlize (version 0.4.16) for chord diagrams, ggtree (version 3.16.0) for phylogenetic tree construction, and corrplot (version 0.95) and igraph (version 1.6.0) for network formation. Additionally, the results plots were generated in R to visualize patterns in AST profiles and gene mutation data across isolates of three groups: CFDC, AMR, and ST.

## Results

### Antibiotic susceptibility

**Figure 2:**
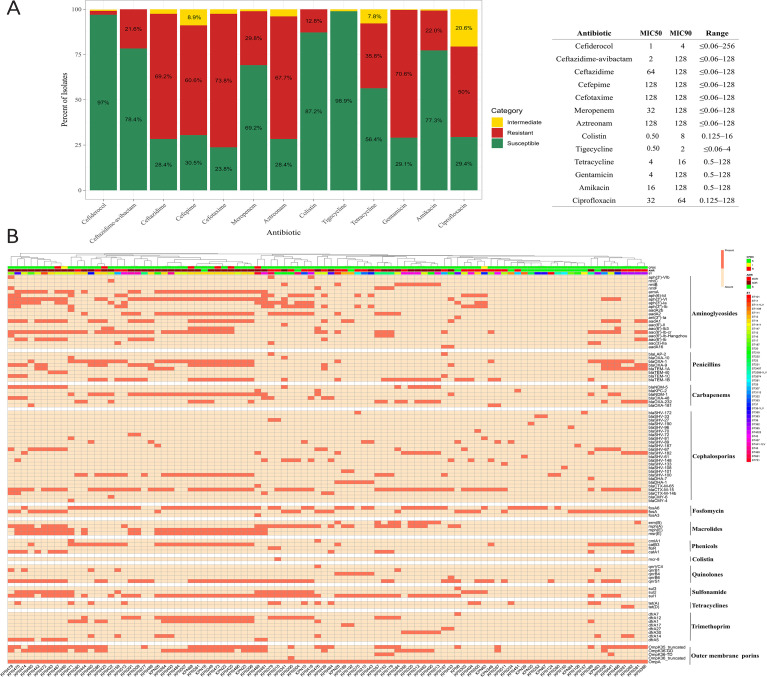
Antibiotic susceptibility phenotypes and genotypes. **(A)** shows the AST profile for all the strains (n=282), with a summary table presenting the MIC_50_, MIC_90_, and MIC range for each antibiotic. **(B)** shows the AMR genes detected in 96 strains selected for WGS. Antibiotic categories, along with a comprehensive list of resistance genes associated with each category, are shown. Additionally, the STs are displayed (color-coded) to reflect the clonality of the strains that have different AMR genes.

CFDC exhibited the highest *in vitro* activity after tigecycline, with a susceptibility rate of 96.9% (MIC_50_/MIC_90_: 0.5/2 μg/ml). The AST results for all 282 strains investigated in this study are summarized in **Figure 2A**, while the full AST data are presented in **Supplementary Data File 1**.

On the basis of the AST results, the strains were classified as susceptible (S) (n=79; 25.9%), MDR (n=132; 43.2%), and XDR (n=71; 23.2%). Moreover, based on the CFDC susceptibility profile, 274 isolates (97.2%) were CFDC-S, 2 isolates (0.7%) were CFDC-I, and 6 isolates (2.1%) were CFDC-R. Based on these findings, 96 samples were selected to represent the resistance phenotype and analyzed by WGS across all resistance categories (16 S, 27 MDR, and 53 XDR) to enable comparative genomic analysis. All 8 CFDC non-susceptible isolates (i.e., 2 CFDC-I, and 6 CFDC-R) were identified as MDR (n=1) and XDR (n=7) and were included in the WGS subset.

### Antimicrobial resistance genes

Analysis of the resistance genes revealed considerable diversity across the isolates, which were clustered on the basis of the ST and AMR profiles (**Figure 2B**; **Supplementary Data File 2**). The mean numbers ± standard deviations of the AMR genes identified in the XDR, MDR, and S isolates were 18.6 ± 4.34, 12 ± 3.90, and 7.4 ± 5.89, respectively. β-lactamase genes were highly prevalent among the isolates, particularly *bla*_CTX-M-15_ (n=62; 64.6%), which was often detected in conjunction with *bla*_OXA-1_ (n=34; 35.4%) and *bla*_TEM-1B_ (n=35; 36.5%). Carbapenemase genes included *bla*_KPC-2_ (n=3; 3.1%), *bla*_NDM-1_ (n=31; 32.3%), *bla*_NDM-5_ (n=17; 17.7%) and *bla*_OXA-48-like_ (n=32; 33.3%). Several isolates harbored more than one carbapenemase gene, such as *bla*_NDM-1_+*bla*_KPC-2_ (n=2; 2.1%), *bla*_NDM-1_+*bla*_OXA-232_ (n=6; 6.3%), *bla*_NDM-1_+*bla*_OXA-48_ (n=1; 1%), *bla*_NDM-5_+*bla*_OXA-181_ (n=1; 1%), *bla*_NDM-5_+*bla*_OXA-181_ (n=6; 6.3%), and *bla*_NDM-5_+*bla*_OXA-48_ (n=3; 3.1%). Co-occurrence analysis revealed that several genes frequently appeared in combination. Notably, *bla*_CTX-M-15_ was often detected in association with carbapenemases (n=45; 46.9%). Carbapenemase genes (*bla*_KPC-2_, *bla*_NDM-1_, *bla*_NDM-5_, *bla*_OXA-181_, *bla*_OXA-232_, and *bla*_OXA-48_) were predominantly identified in ST14 (n=21; 50%), ST147 (n=17; 40.5%) and ST437 (n=8; 19%).

We have also investigated genetic variations in other resistance-associated genes, such as OMP, that influence drug entry. OMPs (*ompK35* and *ompK36*) analysis revealed a substantial number (59.4%) of OMP mutations among the MDR and XDR isolates. Mutations in *ompK36* were present as glycine-aspartate (GD) insertions in 33.3% or as threonine-aspartate (TD) insertions in 4.2% of the isolates, whereas the loss of *ompK36* was detected in only one isolate (KPN290). Additionally, mutations in *ompK35*, such as truncations or reduced identity, were identified in 50% of the isolates, with identities ranging from 6% to 90%. Co-occurrence of both porin types was also detected in 26% of the samples (**Figure 2B****).**

Porin alterations were significantly more prevalent in CFDC non-susceptible isolates. OmpK35 truncations were observed in 66.7% of the resistant isolates compared with 31.5% of the susceptible isolates. Similarly, OmpK36-GD mutations were present in 66.7% of the resistant isolates versus 16.7% of the susceptible isolates. Isolates with truncations in OmpK35 exhibited a 6.2-fold greater mean MIC, whereas those with mutations in OmpK36-GD showed an 8.4-fold greater MIC, suggesting that alterations in porin contribute to variations in the MIC of the CFDC beyond the primary siderophore-mediated uptake mechanism.

### Clonal diversity

**Figure 3:**
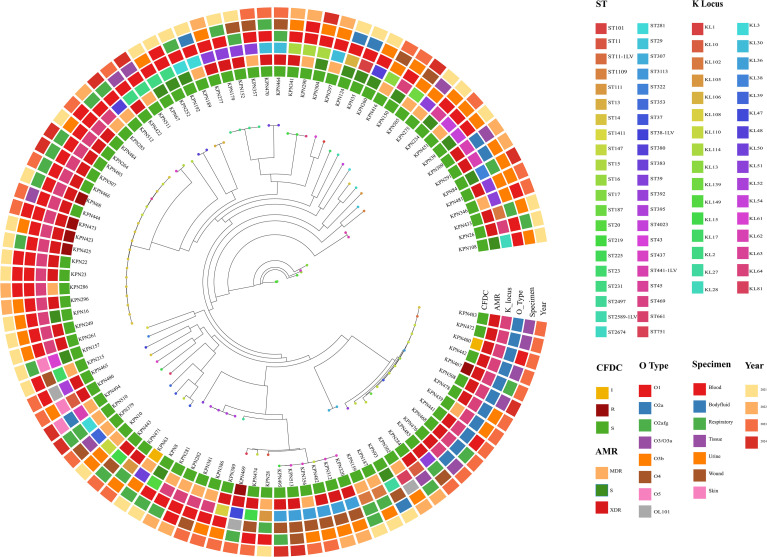
SNP-based maximum-likelihood phylogeny of 96 clinical *Klebsiella pneumoniae* isolates, annotated with AMR phenotype, CFDC susceptibility, sequence type, and antimicrobial resistance gene profile. Concentric heatmap rings denote the following features: year of isolation, source of specimen, K-locus (capsular type), O-antigen type, AMR phenotype (S, MDR, and XDR), and CFDC susceptibility. Clinical sources include blood, body fluid, urine, respiratory samples, wound, tissue, and skin. ST, K-loci, and O-types were determined using in silico tools from genome assemblies.

The core-genome SNP-based phylogeny is shown in **Figure 3**. Clonal diversity was evident across all AMR groups, indicating a broad ST distribution between the groups. That said, certain STs (ST101, ST11, ST23, ST307, ST15, ST395, ST231, ST437, ST14, and ST147), which are recognized as high-risk clones globally, were conserved within drug-resistant isolates. ST147 and ST14 were predominantly linked to the XDR profile, whereas ST231, ST437, and ST395 were restricted to MDR and XDR, and no susceptible isolates were detected amongst them. On the other hand, ST101 and ST23 were exclusive to the S group despite being reported to be high-risk lineages globally. In terms of CFDC susceptibility, ST147 and ST14 also accounted for the majority of the CFDC-R isolates (n=7). This finding was supported by the results of the Shannon diversity analysis, which revealed that the CFDC-R group exhibited the lowest degree of clonal diversity [CFDC-S (3.27), CFDC-I (1.10), and CFDC-R (0.87)]. SNP-based phylogenetic analysis further revealed that the eight CFDC non-susceptible isolates were distributed across phylogenetically distinct lineages. Within ST14, a clonal cluster (KPN423, KPN425, KPN473) was identified, but KPN68 was phylogenetically distinct, suggesting that resistance emerged at least twice independently within this ST. Similarly, within ST147, KPN467 and KPN480 were separated by 41 SNPs, indicating independent emergence. CFDC-R isolates belonging to ST37 (KPN63) and ST751 (KPN469) represent entirely distinct lineages.

OMP profiles revealed a lineage-associated pattern among CFDC non-susceptible isolates. All ST14 isolates displayed concordant OMP alterations, with both OmpK35 truncation and OmpK36-GD mutations present. Similarly, ST147 isolates showed consistent porin mutation patterns within the lineage. However, not all CFDC non-susceptible isolates harbored porin alterations; for instance, KPN63 (ST37) achieved intermediate-level resistance (MIC = 8 µg/mL) despite retaining intact porins. OmpK35 truncations were observed in 66.7% of the CFDC-resistant strains compared with 31.5% of the CFDC-susceptible strains, whereas OmpK36-GD mutations were present in 66.7% of the resistant versus 16.7% of the susceptible isolates. Isolates harboring OmpK35 truncations exhibited a 6.2-fold increase in the MIC, and those with OmpK36-GD mutations showed an 8.4-fold increase. OMP profiles across isolates such as ST14 strains with double porin loss versus ST37 (KPN63) with intact porins both achieve elevated MICs. These findings suggest that porin mutations represent lineage-associated traits that may contribute to CFDC MIC variation in certain high-risk clones but are not required to confer resistance against CFDC.

High-risk isolates of ST14 and ST147 had the highest mean number of acquired AMR genes, at 19.2 ± 5.06 and 18.1 ± 4.29 genes per isolate, respectively. The ESBL gene *bla*_CTX-M-15_ was among the most widespread, detected in 24 isolates and present in 57.1% (24/42) of the STs, including ST14, ST147, ST231, ST307, and ST395 (**Figures 2B**, **3**). Analysis of capsular (K) and lipopolysaccharide (O) antigen types revealed that KL64 was the most common K locus across all resistance groups and was particularly dominant in XDR and MDR isolates, suggesting its strong association with the resistant phenotype. Among the O antigens, O1 was the most prevalent across all the groups, including the S strains, indicating its broad distribution. However, O2a was frequently associated with resistance phenotypes, as it was absent from all the S isolates.

### Mutational patterns within six categories of OS-related genes

In total, 305 mutations were identified across all 6 pathways (**Supplementary Data File 3**), as follows:

#### OxyR system

**Figure 4:**
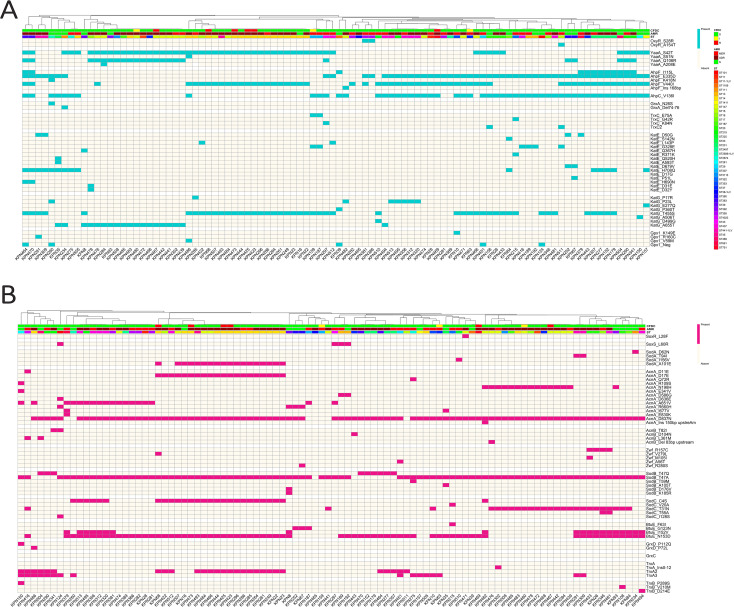
The heatmaps display the presence (colored) or absence (off-white) of amino acid substitutions in detoxification genes regulated by **(A)** the OxyR system and **(B)** the SoxRS system. Each column represents a clinical isolate, clustered by mutation-profile similarity. Top annotations indicate ST, AMR phenotype, and CFDC susceptibility. Rows represent individual mutations grouped by gene.

Notably, 95/96 isolates (99%) carried at least one mutation in OxyR–associated genes, indicating the widespread occurrence of AA substitutions in this pathway across the isolates. Mutations in AhpFC (E335D, V440I, and V136I) were the most common in more than 50% of the isolates. In addition, a mutation in KatG_T455S was highly frequent (49%). One XDR isolate from ST383 (KPN464) exhibited a high number of mutations in *ahpFC* and *katGE* and was gpx1 negative. OxyR, the main regulator of the system, was mutated in 3 MDR isolates (KPN511, KPN381, and KPN386), as shown in **Figure 4A**.

#### SoxRS system

Overall, 99% of the isolates exhibited at least one mutation in the SoxRS system genes. The most consistent mutations observed in this system were TrxB_D324G, AcnA_D837N, and SodB_T47A, which occurred in approximately 90% of the isolates. Another frequent mutation in BtuE_N153D was identified. SoxR, the transcriptional repressor, was mutated (L28F) in one XDR isolate (KPN471) belonging to ST147. Notably, the *trxA* gene was either encoded once and denoted as *trxA* in the map or encoded twice and hence referred to here as *trxA2* and *trxA3*. Mutations in the SoxRS system are shown in **Figure 4B**.

#### Iron regulation

**Figure 5:**
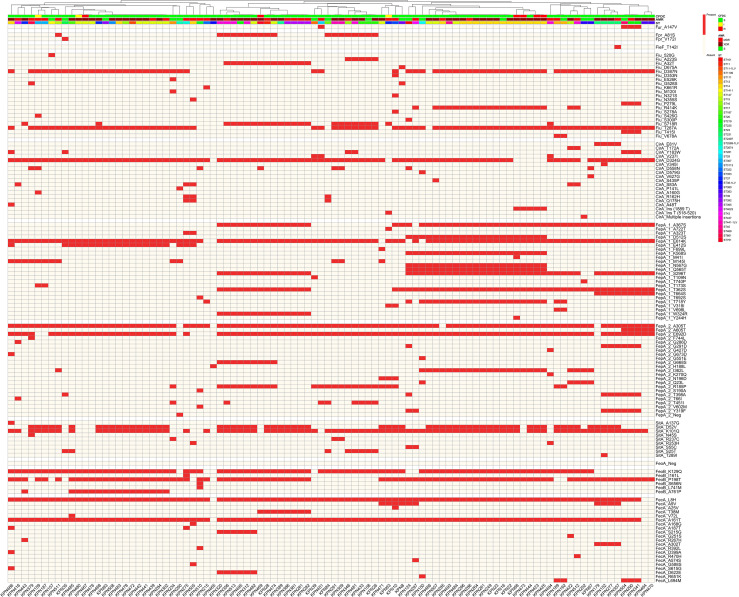
Mutations in iron regulation genes across isolates. The heatmap shows the presence (red) or absence (off-white) of mutations in major genes involved in iron uptake and regulation. Each column represents a clinical isolate, clustered by mutation-profile similarity. Top annotations indicate ST, AMR phenotype, and CFDC susceptibility. Rows represent individual mutations grouped by gene.

As depicted in **Figure 5**, several mutations were identified in all the isolates and were therefore not exclusive to any particular phenotype. These include CirA_D324G, FepA_1_E614K, and FepA_2 (A305T and E692D). Two copies of the enterobactin receptor (*fepA*) were detected and denoted as *fepA_1* and *fepA_2*. The main regulator of iron homeostasis, the *fur* gene, was mutated in only 4.2% of the isolates encompassing the CFDC-S isolates. There were 4 mutations conserved in the CFDC-R isolates, FepA_1_E614K, FepA_2 (A305T and E692D), and FeoB (K129Q and P198T), as well as a T insertion at position 1889, leading to a frameshift mutation in the *cirA* gene. FecA exhibited a slew of AA substitutions, of which L8H and A161T were present in 98% of the isolates.

#### Efflux systems

**Figure 6:**
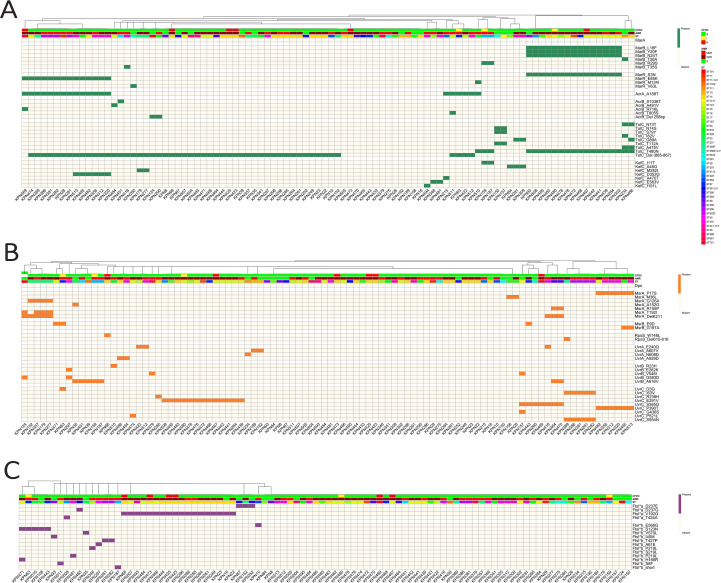
The heatmaps illustrate the presence (colored) or absence (off-white) of amino acid substitutions in genes involved in the **(A)** efflux system, **(B)** DNA repair, and **(C)** penicillin-binding protein (PBP3) category. Each column represents a clinical isolate, clustered by mutation-profile similarity. Top annotations indicate ST, AMR phenotype, and CFDC susceptibility. Rows represent individual mutations grouped by gene.

At least one type of mutation in the efflux genes was detected in 86.5% of the isolates. As depicted in **Figure 6A**, within the AcrAB-tolC system, TolC_T480N was the most frequent variant, whereas in the MarRAB system, MarB (L18F, Y20F, N24T, and S3N) mutations were the most frequent in the MDR and XDR isolates.

#### Repair genes

Repair gene disruptions were noted in 60.4% of the isolates, mostly attributed to the MDR and XDR groups. As depicted in **Figure 6B**, scattered mutations were found across DNA repair genes, with *uvrA*, *uvrC*, *msrA*, and *rpoS* being the most affected. A large subset (40%) of the isolates, mostly drug resistant, did not have any mutations across DNA repair genes. Mutations in *rpoS*, a global stress response sigma factor, were exclusively observed in all CFDC-R isolates. Premature stop codon mutations were identified in two genes, *msrB* and *rpoS*, exclusively in isolates from the XDR group. These nonsense mutations are predicted to result in the formation of truncated proteins, thereby disrupting their function. Specifically, a G deletion in *msrB* at position 197 in KPN495 and KPN513 and a deletion of GG at positions 615–616 in *rpoS* in KPN389 were detected (**Supplementary Data File 4**).

#### PBP-3 genes

Alterations in PBP-3 genes were identified in 37.5% of the isolates, where FtsI*a_V192G was significantly enriched in the CFDC-R isolates (66.7%) compared with the CFDC-S isolates (15.9%) and was predominantly found in the XDR isolates, as depicted in **Figure 6C**.

### Mutation co-occurrence analysis

A comprehensive analysis of 4,278 mutation pairs across six OS-related pathways was performed, with φ coefficient values ranging from -0.851 to 1.000, revealing key pathway-specific patterns. Nearly 52% of mutation pairs exhibited a near-zero correlation (|φ|< 0.15), indicating that a large proportion of mutations arise independently of one another.

**Figure 7:**
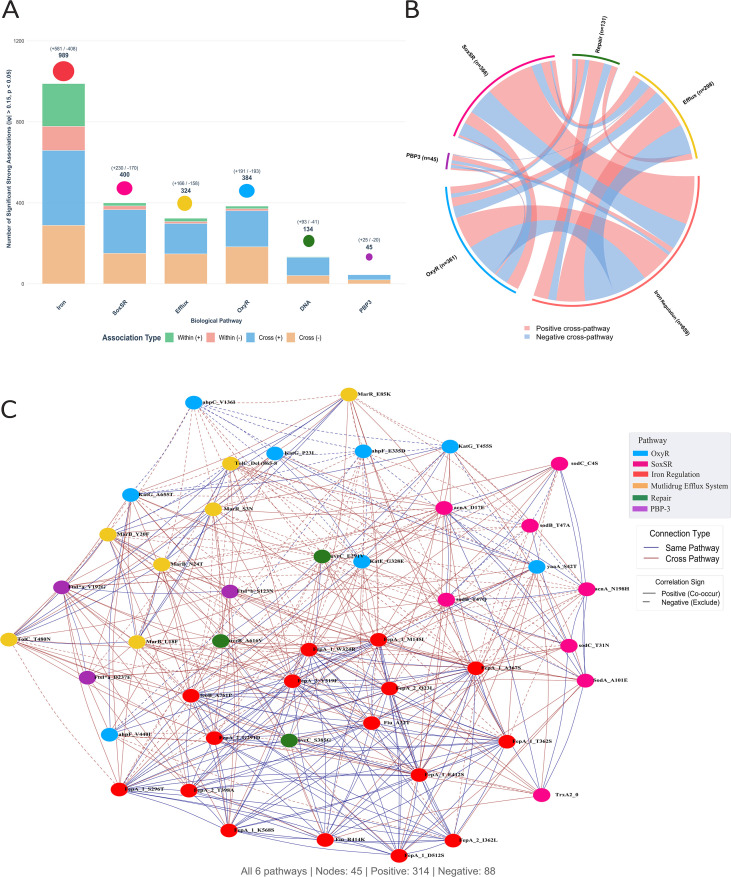
Mutation co-occurrence within the same pathway and across pathways. **(A)** stacked bar chart with overlay circles showing within-pathway and cross-pathway associations. The colored circles above each bar represent the total associations (size proportional), while the numbers in parentheses represent the total positive and negative associations per pathway. **(B)** chord diagram visualization of pathway connections. Bidirectional relationships of pathways with band thickness reflecting interaction strength are shown, while the color indicates whether the association is positive (red) or negative (blue). The numbers in parentheses represent the total strong associations with other pathways. **(C)** network illustration of statistically significant correlations between mutations across different genes. Nodes represent specific mutations, color-coded by functional pathway, while edges indicate positive (solid lines) or negative (dashed lines) correlations.

A total of 1,346 mutation pairs showed significantly strong associations (p <0.05), comprising 772 significant positive (co-occurring) and 574 significant negative (mutually exclusive) correlations. Among the 1,346 significant strong pairs, 416 (30.9%) were within-pathway associations, and 930 (69.1%) were cross-pathway associations, indicating that co-occurring mutations more frequently span different resistance pathways rather than clustering within the same pathway. Mutation co-occurrence within the same pathway and across the six functional OS groups are shown in **Figure 7**, which illustrates the number of strong associations per functional group.

The iron pathway emerged as the central hub with the largest network and the greatest absolute number of significant strong associations (n=989 involving iron-related mutations), followed by the SoxRS, OxyR, efflux, DNA repair, and PBP3 pathways. The PBP-3 pathway was the smallest in terms of the number of mutations and had only one within-pathway pair, limiting the assessment of internal coordination. In contrast, the efflux pathway exhibited the highest rate of within-pathway coordination, with 72.2% of its within-pathway pairs reaching statistical significance, followed by SoxRS (43.6%) and OxyR (34.8%), as shown in **Figure 7A**. The same figure demonstrates that the majority (69.1%) of significantly strong correlations were cross-pathway interactions, while 30.9% were within-pathway interactions. The cross-pathway interactions are shown in **Figure 7B**. Among the significant cross-pathway associations, positive interactions were moderately more frequent than negative interactions (514 positive vs. 416 negative, representing a 1.2:1 ratio and 55.3% positive). Iron appeared as a central hub, participating in the three largest cross-pathway interaction sets (Iron–SoxRS: 215 significant pairs, Iron–OxyR: 189, and Efflux–Iron: 166). The strong directionality of the DNA–efflux cross-pathway pair was notable, with 17 positive versus only 1 negative significant association, suggesting highly cooperative interactions between DNA repair and efflux systems. The SoxRS–OxyR interaction was nearly balanced between positive (n=36) and negative (n=37) significant associations, indicating both cooperative and competitive interactions between these two OS response pathways. This balance suggests context-dependent selection of stress responses, where the two systems may act synergistically under some conditions and antagonistically under others.

The next step was to identify individual mutations from each gene category that were strongly correlated and occurred simultaneously with other mutations. Network correlation analysis (**Figure 7C**) revealed the most connected mutations in the network. AhpC_V136I (OxyR pathway) was the most highly connected mutation, with 58 edges spanning all six pathways, followed by Fiu_D387N, FepA_1_S296T, FepA_1_T362S, AhpF_E335D, and TolC_Del. Dense intra-pathway connectivity was observed among iron-uptake genes, particularly among *fepA, fiu*, and *fecA* variants. Among individual mutations, FecA_A9V showed strong positive correlations with several iron pathway variants, including FepA_2_Y319F, FepA_2_G291D, and FepA_2_T398A, as well as cross-pathway links to the DNA repair mutation MsrA_K211Del, the PBP3 mutation FtsI*b_S123N, and the SoxRS mutation SodB_T47Q. Additionally, YaaA_S42T, identified primarily in the CFDC-R group, was positively correlated with Fiu_D387N, Fiu_T287A, and AcnA_D17E, thereby linking OxyR genes to iron acquisition and the SoxRS pathway. In contrast, YaaA_S42T was negatively correlated with AhpF_E335D, AcrA_A188T, FepA_1_W324R, MarR_E85K, and FepA_1_W324R, indicating mutations in the OxyR, efflux, and iron pathways. Contrary to initial expectations, the efflux-related gene *tolC* was not isolated within the network. The TolC_Del (865–867) variant appeared to be among the most connected mutations, showing significant associations with mutations from all six pathways, while TolC_T480N also exhibited broad connectivity. TolC variants were particularly linked to iron pathway mutations, including strong positive correlations with FepA_1_T362S, FepA_1_A367S, and FepA_1_S296T, as shown in **Figure 7C**.

### Impact of individual and co-occurring mutations on CFDC MIC

**Figure 8:**
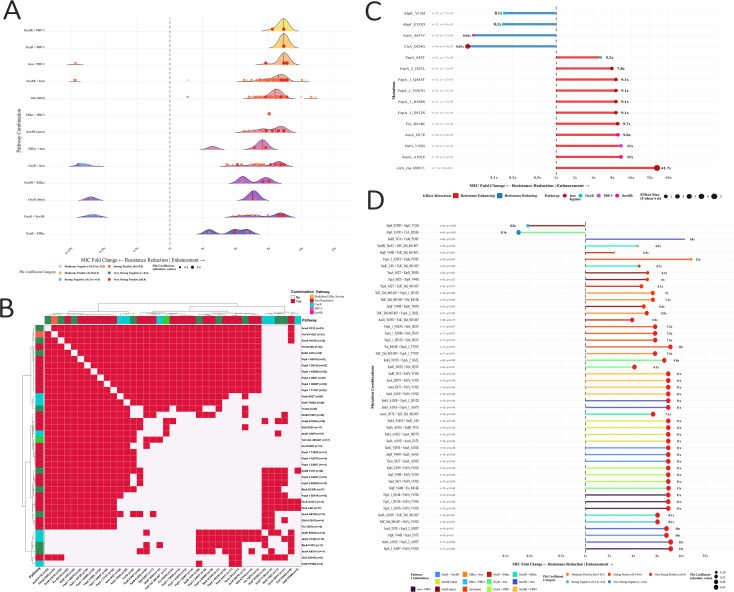
Mutations with a significant impact on CFDC suppressibility. **(A)** Ridge plot of pathways showing the distribution curves for each pathway combination with density patterns across all combinations. The impact on the MIC is shown with phi coefficient color coding (red = very strong, blue = weak). **(B)** Heatmap showing different combinations of mutations with significant effects on the CFDC MIC. **(C, D)** single mutation and mutation combinations by effect magnitude with distribution across pathways. The top 5 mutation combinations per pathway are shown. The horizontal bars represent the fold change in the MIC, with the point size reflecting the strength effect (Cohen’s d and the phi coefficient for single and double mutations, respectively). The color coding shows the pathway or mutation type. The numbers of isolates with the specified mutation are also shown.

Overlaying the network findings with the phenotypic data revealed that several mutations are correlated with the MICs of CFDC. Association analysis revealed that 15 individual mutations and 402 pairs of co-occurring mutations significantly affected the CFDC MIC (**Figure 8**). Strong positive correlations (φ>0.15) were detected in 352 combinations (87.6%), whereas strong negative correlations (φ<-0.15) were detected in 50 combinations (12.4%). Cross-pathway synergies dominate, with the top combinations showing increases of ≥10x MIC up to 21x MIC (**Figures 8A, D**). The iron pathway was the major hub for resistance combinations, while PBP-3 combinations were less frequent but still significant. Iron intrapathway mutation combinations dominate. The cross-pathway interactions are significant, with consistently strong effects (all φ> 0.15). Iron pathway mutations were the major contributors, along with OS pathway mutations, namely SoxRS and OxyR.

**Figure 8C** shows 15 individual mutations associated with a significant change in the CFDC MIC. Most of them (n=11) were associated with increased MICs, while only 4 mutations were associated with reduced susceptibility. It is noteworthy that 14/15 individual mutations (except *cirA*_Ins1889T) associated with a significant impact on the CFDC MIC frequently co-occurred in multiple combinations, as shown in the heatmap (**Figure 8B****),** clearly depicting the interaction between various pathways and the co-occurrence of a set of mutations in various combinations. The most frequent significant mutations include SodA_A101E, acnA_D17E and FtsI_V192G*a (each appearing in 33 combinations), followed by the iron pathway cluster (in 32 combinations), which includes Fiu_R414K, FepA_1_D512S/K568S/N567G/Q565T, and FepA_2_I362L, ultimately YaaA_S42T (28 combinations), among other less frequent mutations. Examples of the top 5 combinations from each of the 13 types of pathway interactions, along with their impact on MICs, are shown in **Figure 8D**.

Among the individual mutations with the strongest phenotypic associations, FeoB_K129Q, FepA_2_E692D, SodB_T47A, FecA_L8H, FecA_A161T, and AcnA_D837N were consistently found in all the CFDC-R isolates and were significantly associated with increased MICs. Conversely, mutations such as Fur_A147V, Fiu_P279L, AhpF_E335D, UvrB_A616V, CirA_Y183W, CirA_S83A, and Zwf_R157C were linked to decreased CFDC MICs. Additionally, several impactful mutations, such as CirA_Ins1889T, FeoB_P198T, FepA_2_A305T, and SodB_T47A, demonstrated strong individual associations with increased MICs, despite being excluded from the network because of the threshold criteria.

### Category-level mutation by the CFDC susceptibility, AMR, and ST groups

**Figure 9:**
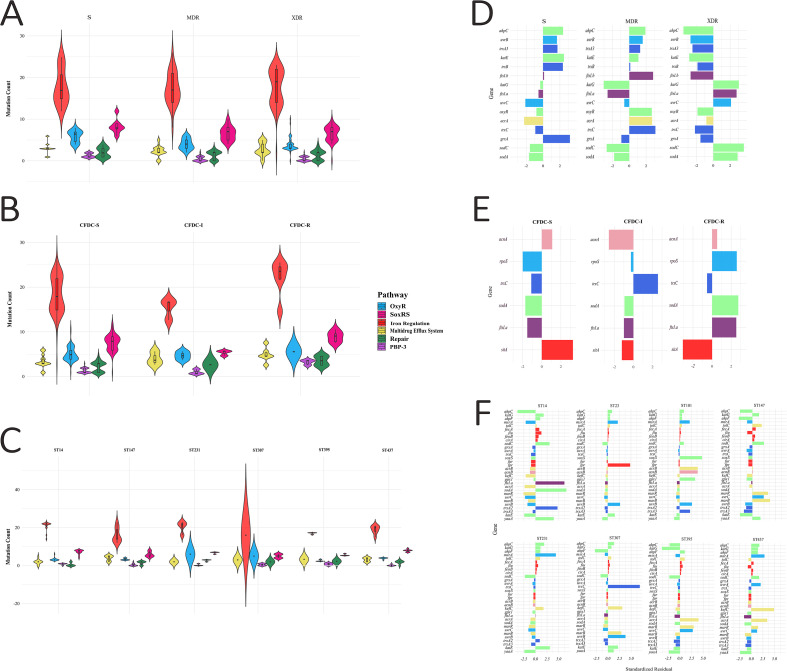
Distribution and statistical comparison of mutation burden across functional categories. **(A–C)** violin plots display the total number of mutations associated with different categories. **(D–F)** standardized residuals from chi-square analyses of significant gene-level mutations.

Violin plots were constructed to illustrate the spread and density of mutation counts across the categories stratified by the AMR, CFDC susceptibility, and ST groups. Iron regulation resulted in a broader distribution of mutation burden, as depicted in **Figures 9A–C**.

To investigate the distribution of mutations in OS genes across clinical groups (CFDC, AMR, and ST) within each category, the Kruskal–Wallis test and Chi–square test were employed. The results of the Kruskal–Wallis test revealed that the mutation burden in the ST group consistently varied across all OS categories, including OxyR, PBP-3, DNA repair, iron regulation, SoxRS, and efflux system (p < 0.05), with ST147 carrying the highest load of mutations. The OxyR pathway showed a significant burden across AMR groups (*p=0.003*), with a higher burden in MDR isolates.

Moreover, Chi-square analysis was performed to assess the association between category mutations and the groups. Similar to the Kruskal–Wallis analysis, the ST group showed the strongest association with mutations across all pathways. The efflux system was most strongly associated with the ST (*p<0.0003*). Among the AMR group, significant associations were observed for PBP-3 (*p<0.01*) and OxyR (*p<0.01*), indicating that mutations in these systems were unevenly distributed across resistance categories, with the highest contributors belonging to the XDR and S groups, respectively. The other categories were not significantly associated with AMR (*p>0.05*). The CFDC group was significantly associated with PBP-3 mutations (*p=0.05*), whereas the frequency of mutations was highest in the CFDC-R group.

### Gene-level mutations by the CFDC susceptibility, AMR, and ST groups

**Figures 9D–F** illustrates the gene-level associations with AMR, CFDC susceptibility, and STs, as determined by standardized residual analyses. Mutation data were further categorized into eight mechanistic pathways, namely, detoxification, iron regulation, PBP-3, efflux system, redox homeostasis, tricarboxylic acid (TCA) cycle, DNA repair, and pentose phosphate pathway (PPP), to improve biological interpretation and eliminate redundancy among functionally overlapping genes. OxyR (*p=0.008*) mutation was also significantly increased in MDR isolates. XDR isolates presented enriched mutations in the *ftsI*, *sodC*, *sodA*, and *marA* genes. Analysis revealed that gene mutations highlighted four genes (*fepA*, *sodA*, *rpoS*, and *trxC*) as being significantly associated with CFDC susceptibility and predominantly enriched in CFDC non-susceptible isolates (**Figure 9E**). In terms of clonal distribution, several genes were significantly associated with the high-risk ST group (**Figure 9F**). The strongest association was observed between ST231 and ST383 and the *msrA* gene (*p<0.0001*). ST395 was enriched in mutations in the DNA repair gene *uvrC* and the efflux gene *acrA* (p=0.004). Mutations in the *yaaA* gene were enriched in isolates belonging to ST231, ST395, and ST437 but markedly depleted in other high-risk lineages, such as ST14 and ST147 (*p<0.001*). High-risk clones, including ST231, ST147, ST14, ST307, and ST395, exhibit strong clonal enrichment of mutations in detoxification and efflux system genes.

Several genes showed significant associations in at least two groups. SodA was significantly enriched in both the CFDC-R (*p<0.03*) and XDR (*p=0.03*) isolates, whereas *ahpC* had significantly fewer mutations in both the XDR (*p<0.001*) and ST14 (*p<0.003*) isolates than expected. Interestingly, mutations in the efflux genes *acrA* and *marB* were significantly associated with MDR and XDR isolates (p < 0.01) and were enriched in the high-risk clones ST395 (p < 0.004) and ST147 (p < *0.001*). Mutations in the DNA repair gene uvrB_A616V were enriched in ST307 (p<0.0001). Finally, *fepA* gene mutation was significantly enriched in both the CFDC-R isolates and ST14 (*p<0.001*).

### Specific gene mutation level

**Figure 10:**
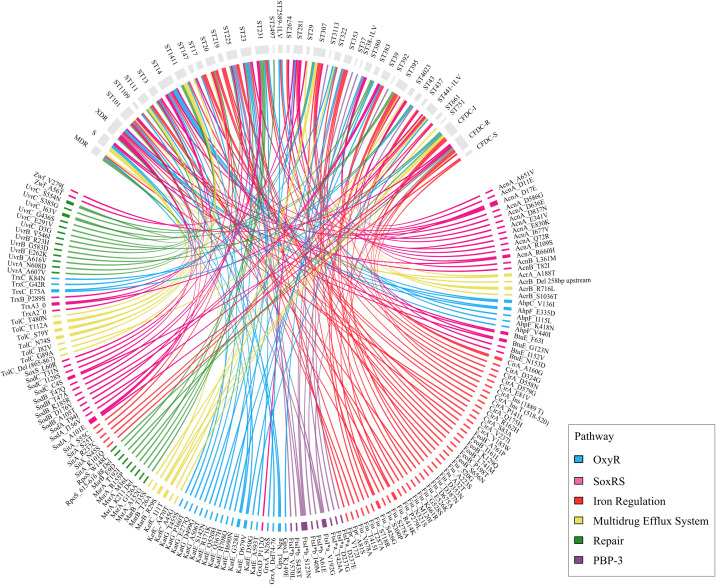
Chord diagram illustrating the association between sequence type STs and resistance with significant mutations in oxidative stress pathways across isolates. Each ribbon connects a specific ST/resistance phenotype (top arc) with a gene mutation (bottom arc). The thickness in the outer ring represents the frequency of mutations per gene, ST and resistance phenotype.

Targeted analysis of individual mutations revealed several mutations that were strongly associated with high-risk clones and resistance phenotypes (**Figure 10**). Within the OxyR regulon, the *gpx1* gene was negative in ST101 (*p* < 0.0001), whereas the catalase gene mutation KatE_D679V was enriched in the S group (*p* < 0.01). Although AhpC_V136I and AhpF_E335D were significantly associated with the AMR group (p<0.02), their frequencies were lower in XDR isolates (37% and 41%, respectively) than in MDR (69% and 66%) and susceptible isolates (81% and 75%), and they were negatively associated with ST14 (*p<0.003*). The superoxide dismutase gene SodA_A101E was significantly associated with both XDR (p=0.017) and CFDC-R (p=0.006), with higher frequencies observed in the CFDC-R isolates (66.7%) than in the CFDC-S isolates (15.9%). Other mutations were depleted in the CFDC-R gene, such as AcnA_D17E (*p=0.01*) and SodC_C4S (*p<0.05*). Multiple mutations in Fiu (e.g., Fiu_D675A, Fiu_G528S, Fiu_S300P, and Fiu_K661R) and CirA (e.g., CirA_E81V, D579G, A160G, and P141L) were enriched across different STs, such as ST39, ST101, ST111, ST231, ST307, and ST353. Mutations in the *fecA* gene were associated with high-risk STs but not with the AMR or CFDC groups. Additionally, *cirA*_Ins (1889 T) was highly enriched in the CFDC-R isolates (*p<0.0001*), whereas CirA_S83A was enriched in the MDR isolates (*p<0.04*), indicating a strong link between CirA disruption and the resistance phenotype. Efflux system mutations were distinctly associated with the ST group. For example, MarB_R29S and KefC_I11T were enriched in ST307 (*p<0.001*), and TolC_T480N was enriched in ST147. In contrast, TolC_N74S, TolC_T112A, and TolC_S79Y were significantly enriched in S isolates (*p=0.006*), indicating that dysfunctional efflux pumps lead to a susceptible phenotype. Multiple DNA repair–associated mutations in *uvrA*, *uvrB*, *uvrC*, *msrA*, *msrB*, and *rpoS* resulted in strong clonal enrichment, accompanied by resistance phenotypes. These include RpoS_615–616 Del in ST2497, UvrB_E262K in ST43, and MsrA_T192I in ST231. Multiple UvrC mutations (e.g., S554N, G436S, and I63V) were enriched in ST395, whereas UvrB_A616V was enriched in ST307 (*p<0.0001*), whereas UvrC_I63V and UvrC_S554N were enriched in MDR isolates (*p<0.04*). Finally, FtsI_V192G*a was highly enriched in the CFDC-R, XDR, and ST14 isolates (*p<0.01*).

### Correlation analysis

**Figure 11:**
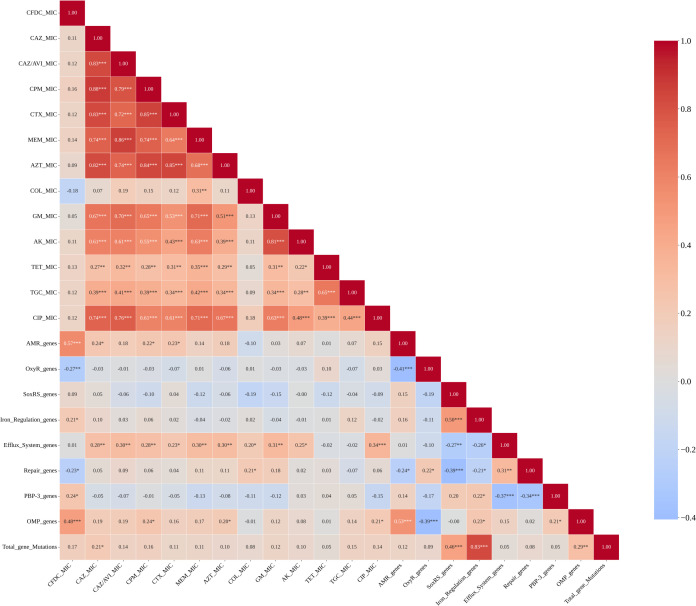
Correlations between the number of mutations in OxyR, SoxRS, the efflux system, DNA repair, iron regulation, PBP3, outer membrane porin (OMP), and the total number of mutations with the MICs of antibiotics and the number of AMR genes. *p value < 0.05, **p value < 0.01, ***p value < 0.001.

To further explore the interplay between OS pathways with the MIC of CFDC and AMR genes, we conducted a Spearman correlation analysis across all categories, as shown in **Figure 11**. The number of OxyR mutations exhibited a non-significant negative correlation with the MIC values across all antibiotics, except for that of the CFDC, which showed a significant negative correlation with mutation in the pathway (ρ = –0.264, *p < 0.01*). The number of DNA repair gene mutations also strongly negatively correlated with the CFDC MIC (ρ = –0.239, *p < 0.01*). Moreover, only CFDC was significantly correlated with the number of mutations in iron regulation genes (ρ = 0.201, *p = 0.050*). In contrast, no other antibiotic was significantly associated with this pathway. The efflux-related genes strongly positively correlated with the MICs of most antibiotics, particularly β-lactams and aminoglycosides, such as CAZ, CAZ/AVI, CPM, MEM, AZT, GM, AK, and CIP, but no significant correlation was found with the concentrations of CFDC or tetracyclines (TET and TGC). Moreover, a strong positive correlation (ρ = 0.443, *p < 0.001*) was found between the number of PBP-3 mutations and the MIC of the CFDC, further indicating the effect of PBP-3 mutations on CFDC susceptibility.

Strong positive correlations were detected between the AMR gene count and mutations in OMPs (ρ = 0.53; *p < 0.01*) and PBP-3 (ρ = 0.44; *p < 0.01*), and the number of mutations in OxyR genes was negatively correlated. Additionally, there was a significant positive correlation between OMP mutation and the MIC of the CFDC as well as the number of AMR genes (ρ = 0.488 and ρ = 0.529, respectively, with *p < 0.001*). In addition, the AMR gene count was correlated with PBP-3 gene mutation (ρ = 0.438, *p < 0.00001*). On the other hand, OxyR gene mutation (ρ = –0.406; *p < 0.0001*) and DNA repair pathway mutation (ρ = –0.237; *p = 0.02*) were negatively correlated, indicating that isolates harboring more OxyR and repair gene mutations were associated with fewer AMR genes. No associations were detected between the AMR gene count and SoxRS, efflux, or iron regulation genes.

## Discussion

The increasing prevalence of CFDC resistance necessitates in-depth investigations into the mechanisms underlying it (Lan et al., 2022; Polani et al., 2025). Most studies have focused on the effects of β-lactamases, particularly carbapenemases, on the efficacy of CFDC; however, the role of iron that accompanies CFDC entry and its contribution to oxidative toxicity have been rarely explored. Iron overload, particularly during disrupted homeostasis, can exacerbate the damaging effects of OS via the Fenton reaction (Jones, 2007; Borisov et al., 2021). This study presents a comprehensive genomic investigation of 51 OS-related genes in KPN and their relevance to AMR phenotypes, CFDC susceptibility, and clonal lineages by coupling WGS with detailed mutational profiling across 96 well-characterized clinical isolates.

Consistent with previous reports, our strains were highly resistant to most β-lactams, while CFDC retained the highest *in vitro* activity (Stracquadanio et al., 2021; Falcone et al., 2022). MDR and XDR strains harbored the greatest number of AMR genes, with the progressive accumulation of resistance determinants reflecting the genomic complexity characteristic of high-risk KPN lineages (Pitout et al., 2015). AMR gene profiling revealed a predominance of *bla*_CTX-M-15_ in both MDR and XDR isolates. Additionally, carbapenemase genes, including *bla*_NDM-1_, *bla*_NDM-5_, *bla*_OXA-181_, *bla*_OXA-232_, and *bla*_KPC-2_, were detected in more than 50% of the isolates, whereas several isolates co-harbored multiple carbapenemase genes, highlighting the alarming spread of carbapenemases in the UAE (Ahmed et al., 2024). The progressive accumulation of AMR genes across different *Klebsiella* isolates reflects the genomic complexity characteristic of high-risk lineages, which is consistent with findings from clinical KPN studies in other settings (Sahayarayan et al., 2024). The presence of *bla*_NDM_ variants, particularly *bla*_NDM-5_, is of critical concern given recent findings showing its ability to enhance resistance to CFDC (Poole, 2012; Dam et al., 2018; Al-Marzooq et al., 2025a), especially in co-occurrence with other carbapenemase genes and *cirA* mutations (Lan et al., 2022; Simner et al., 2022; Mezcord et al., 2024; Al-Marzooq et al., 2025a). Our data corroborates this mechanism, as all CFDC-R isolates harbored *bla*_NDM_ and a frameshift insertion in *cirA* (T1889Ins), impairing CFDC uptake and increasing drug degradation. Several MDR and XDR strains are global high-risk clones, such as ST14, ST147, and ST437, which consistently carry multiple AMR genes and have been implicated in multiple outbreaks, posing serious challenges to public health systems (Turumtay et al., 2022; Lobato et al., 2023; Al-Zahrani et al., 2024). Notably, while CFDC-R KPN strains reported worldwide belong to ST383, ST11, and ST147 (Moon and Huang, 2023; Liu et al., 2024), our CFDC-R isolates belong to global high-risk clones (ST14, ST147), and other internationally distributed clones, such as ST37 and ST751. The specific clonal distribution reflects the regional epidemiology and underscores the broader relevance of our isolate collection to global epidemiology. Noteworthy, the reported OS pathway mechanisms are intrinsic to KPN and are not clone-specific. Also, the genes investigated are conserved in the species, which suggests that the link of iron regulation, OS response, and CFDC susceptibility is likely broadly applicable beyond the UAE population. Similar OS pathway adaptations may exist in globally prevalent high-risk clones such as ST383 and ST11, which are not dominant in our collection, and their contribution to CFDC susceptibility warrants investigation in other geographic settings.

Our comprehensive analysis revealed a multi-level resistance architecture. A total of 305 mutations were identified, with individual mutations providing baseline CFDC resistance and epistatic combinations, resulting in high-level resistance of up to a 21x increase in the MIC. The distribution of these mutations across resistance phenotypes and clonal lineages revealed a multi-pathway resistance architecture, detailed in the following sections.

CFDC entry into bacterial cells relies on two distinct routes (**Figure 1**), passively through the outer membrane porins (OmpK35/OmpK36) and actively via siderophore uptake channels/receptors TonB-dependent iron receptors (CirA, FepA) (Giacobbe et al., 2020). OMP mutations were prevalent among the MDR and XDR isolates. OmpK36 loop 3 insertions, including the TD variant detected in our isolates, have been associated with porin channel constriction and reduced antibiotic permeability (Wong et al., 2019; David et al., 2022). This mechanism may contribute to the observed association between these mutations and increased CFDC MIC values, suggesting reduced drug entry through this route. With respect to the siderophore-mediated route, we identified several mutations in CirA receptor, for example, an insertion mutation at position 1889 in *cirA*, a mutation that was mostly found in CFDC-R isolates and statistically associated with resistance, possibly due to the disruption of CirA-mediated drug uptake. Mutations in FepA (E614K, A305T, E692D) and FeoB (K129Q, P198T) were also conserved in CFDC-R isolates from high-risk clones such as ST14, further suggesting that *K. pneumoniae* may develop resistance to CFDC through cumulative alteration in the iron-mediated uptake system. These findings were further supported by the mutation correlation analysis, which revealed dense co-occurrence among the iron acquisition genes *fecA*, *fepA*, and *feoB*, along with positive associations with the CFDC MIC. The downregulation of the *feoA* and *feoB* genes has been linked to CFDC resistance in *Acinetobacter baumannii*; however, this has not been reported in *K. pneumoiae* (Yukun, 2022). The number of mutations in iron-regulating genes was significantly and positively correlated with the CFDC MIC, further supporting the significance of functional iron uptake genes for the efficient uptake of CFDC. Previously, the FecA_G569A substitution was reported in CFDC-R KPN isolates (Padovani, 2023). This variation was not detected among our strains, which had several types of *fecA* mutations, such as FecA_L8H and FecA_A161T, that were significantly correlated with elevated MICs. We previously reported that overexpression of the *fecA* gene was detected in isolates with higher CFDC MICs, although *fecA* mutations were not investigated (Daoud et al., 2022). Overall, these findings support the hypothesis that a defective iron transport system affects the CFDC susceptibility pattern. The co-occurrence of porin mutations and iron receptor disruptions in CFDC-R isolates, particularly in ST14, suggests that these two mechanisms act additively to reduce intracellular drug accumulation. Some isolates (KPN63) achieved elevated MICs despite intact porins, indicating that disruption of the siderophore uptake route alone can be sufficient to confer resistance. However, the precise mechanistic interaction between these two entry routes remains to be fully investigated.

It is well established that iron overload enhances ROS production via the Fenton reaction (Andrews et al., 2003). CFDC treatment has been suggested to be associated with elevated intracellular iron and ROS levels (Du et al., 2022), although this observation warrants further investigation. OS response systems in KPN comprise three major systems OxyR, SoxRS, and MarRAB, and mutations across these systems were distributed among isolates with various resistance phenotypes. The OxyR regulon is a redox-sensitive transcription factor primarily activated by H_2_O_2_, which leads to the expression of key detoxification genes, including *katE*, *katG*, and *ahpCF*. Mutations in this region have resulted in diminished detoxifying enzyme activity, and deletion of *oxyR* in KPN has been shown to improve antibiotic susceptibility (Lee et al., 2004; Huang et al., 2013; Srinivasan et al., 2013). We identified mutations in detoxification genes such as SodB_T47A, AhpC_V136I, and KatG_T455S that were significantly associated with increase in the CFDC MIC in our study, supporting the role for OS adaptation in modulating CFDC susceptibility.

Within the OxyR system, AhpC_V136I and AhpF_E335D were significantly associated with the AMR group but depleted in XDR isolates, suggesting that these detoxification variants are progressively lost as resistance intensifies (Liu et al., 2018). Conversely, KatE_D679V was enriched in susceptible strains, consistent with a loss-of-function mutation that increases susceptibility to OS damage. OS response pathways, including OxyR-mediated regulation, play a central role in bacterial adaptation to ROS (Huang et al., 2013; Anand et al., 2020). A significant negative correlation was observed between mutations in OxyR and DNA repair genes and CFDC MIC, indicating that impairment of detoxification and repair systems enhances CFDC efficacy. In isolates with intact OS response systems, ROS can be effectively neutralized, limiting cellular damage and resulting in higher MIC values. In contrast, mutations in these pathways may lead to intracellular ROS accumulation, increasing vulnerability to CFDC-induced OS and bacterial killing, thereby lowering MICs (Diaz-Diaz et al., 2021; Bhattacharya et al., 2025). Furthermore, while DNA repair systems normally mitigate ROS-induced damage, impairment of these pathways reduces the bacterial ability to recover from oxidative injury, thereby further increasing CFDC susceptibility. Additionally, *yaaA* gene, which reduces free intracellular iron to limit Fenton reaction-mediated ROS generation, was found to be negatively associated with high-risk clones ST14, ST147, and ST307 (Liu et al., 2011). The conservation of wild-type *yaaA* in these clones may reflect an advantage under iron stress conditions, contributing to their global dissemination. Together, these findings support our hypothesis that CFDC exerts part of its bactericidal activity through iron-mediated OS, with OS pathway integrity serving as a key factor in drug susceptibility.

Efflux pump systems play dual roles in OS adaptation and multidrug resistance, activated by both antibiotic treatment and ROS accumulation via MarR oxidation and downstream *marA* induction (Levy, 2002; Bratu et al., 2009). Mutations in the *acrA*, *acrB*, *marA*, *marB*, and *tolC* efflux genes were strongly clonal, particularly in high-risk clones ST307 and ST147, in agreement with previous reports (Dey et al., 2023). Conversely, TolC mutations (N74S, T112A, and S79Y) were significantly more common in susceptible isolates. Gene mutations impairing efflux function are reported to increase susceptibility to antibiotics (Robert L, 2020). The association of *marB* mutations with MDR and XDR phenotypes is consistent with the established role of the MarRAB system in multidrug efflux, despite a lack of reports on the effects of *marB* mutations (Piddock, 2006). However, whether these mutations result in upregulation, repression, or altered substrate specificity remains unclear. The SoxRS regulon, activated by superoxide anions via SoxR oxidation, regulates genes involved in OS defense, DNA repair, redox balance, and efflux (Koutsolioutsou, 2005). In our study, although *soxR* mutations were rare and were identified only in one XDR isolate (ST147), several SoxRS-regulated genes were associated with resistance phenotypes. Specifically, *sodA* and *marA* mutations were frequent among XDR and CFDC-R isolates. However, the overall mutation burden in the SoxRS system was not significantly associated with susceptibility to CFDC or AMR. These findings suggest that although some SoxRS genes may influence resistance, the regulon as a whole does not predict susceptibility to CFDC.

Mutations in *rpoS*, a global stress sigma factor, were observed only in the CFDC-R isolates. Mutations in the *rpoS* gene were shown to improve the survival of bacteria and increase virulence (Duan et al., 2021). Likewise, enriched mutations in uvrC in ST395 and MsrA_T192I in ST231 highlights lineage-specific accumulation of repair defects. Interestingly, several DNA repair mutations (e.g., UvrC_I63V and UvrC_S554N) were associated with MDR but not XDR isolates. These findings suggest that MDR strains exhibit moderate tolerance to ROS damage and contribute to early resistance evolution, whereas highly resistant strains rely more strongly on the efflux system. Furthermore, activation of the SOS response in response to DNA damage can trigger error-prone DNA repair mechanisms, promoting new resistance mutations (Crane et al., 2021). These findings align with our network analysis, which revealed that mutations in *rpoS*, *sodA*, *trxC*, and *fepA* form a subset of highly connected nodes across the resistance phenotype, CFDC susceptibility, and STs. Their recurrent presence in high-impact combinations further supports their potential as predictive biomarkers for resistance development.

Pathway ranking reveals the central position of iron, which serves as a coordinator in the resistance network, with the highest internal coherence and strongest cross-pathway interactions. While cooperation dominated the resistance network, specific negative associations were observed, particularly between OS pathways. OS pathways (SoxRS/OxyR) have emerged as potential targets for exploiting competitive dynamics. Efflux systems represent optimal combination therapy targets because they have the highest cooperation rates. Notably, the PBP-3 pathway operates independently of broader network coordination, indicating that PBP-3 mutations are not functionally linked to OS response pathways.

CFDC resistance emerges through coordinated multi-pathway networks rather than a single gene or mechanism, with drug acquisition and OS and iron homeostasis serving as the central hub. The central position of iron is biologically consistent with its role in bacterial physiology as well as its role in aiding the hijack of iron uptake receptors CirA and FepA for entry. Intracellular iron drives ROS generation via the Fenton reaction linking iron homeostasis directly to OS response pathways, and key TCA cycle enzymes AcnA and AcnB are iron-sulfur proteins sensitive to OS damage. Iron starvation and OS signals modulate efflux pump expression through SoxS and MarA (Zheng et al., 1999). Similarly, the *yaaA* gene that reduces free intracellular iron to limit the Fenton reaction-mediated ROS generation further illustrates how iron homeostasis genes sit at the interface of oxidative damage control (Liu et al., 2011). Cross-pathway interactions showed a near-equal balance of positive and negative associations, reflecting both cooperative and competitive dynamics. Critically, the iron entering the cell complexed with CFDC is not only a facilitator of drug uptake but an active contributor to its bactericidal activity by amplifying intracellular ROS generation and intensifying OS damage.

Several genes*, sodA, trxC, fepA, and rpoS* that were significantly associated in CFDC non-susceptible group, AMR classification, and STs, intersecting OS response, metal homeostasis, and global transcriptional regulation, represent candidate biomarkers for the CFDC resistance phenotype in KPN. A key limitation of this study is the small number of CFDC non-susceptible isolates (n=8), which limits the strength of subgroup comparisons. This was partially addressed by using Spearman correlation analysis on absolute MIC values rather than resistance categories alone, capturing susceptibility variation even among phenotypically susceptible strains. Functional validation through isogenic knockouts, allelic replacement mutants, and complementation experiments remains necessary to confirm whether the identified mutations directly affect CFDC susceptibility, and future multiomics studies integrating transcriptomics and proteomics with genomics will be essential to confirm their functional significance. The hypothesized mechanistic model of CFDC antibacterial activity based on our findings is shown in **Figure 1**.

## Conclusions

This study reveals a complex interplay among OS pathway mutations, AMR, and CFDC susceptibility in *K. pneumoniae*. High-risk clones harbor accumulated mutations spanning detoxification, iron acquisition, efflux, and DNA repair pathways, reflecting multifaceted adaptations to both antibiotic- and host-imposed OS. CFDC resistance in KPN is multifactorial, involving the collaborative effects of β-lactamase production (particularly *bla*_NDM_), mutations in iron transport genes that impede siderophore-mediated drug entry, and efflux/porin alterations that reduce intracellular drug accumulation. The significant inverse correlation between detoxification/repair mutations and the MICs of CFDC provides mechanistic insight into the drug’s mode of action, suggesting that in addition to inhibiting cell wall synthesis, CFDC may exert a secondary bactericidal effect through iron-mediated oxidative damage. Specifically, the catechol siderophore moiety of CFDC may increase intracellular iron levels, promoting ROS generation via the Fenton reaction and intensifying OS. This proposed dual mechanism is supported by our finding that unique OS pathway mutation profiles correlate with reduced CFDC susceptibility, with iron regulation genes serving as a central hub coordinating cross-pathway interactions. The identification of clone-associated co-mutation networks spanning iron uptake, OS response, and efflux systems offers candidate genomic markers for resistance surveillance and diagnostic development. However, experimental validation through multiomics approaches is warranted to confirm the functional and clinical significance of these associations. Nonetheless, the OS pathway genes identified here represent promising new targets for surveillance and potential therapeutic intervention in the fight against drug-resistant KPN.

## Data Availability

The datasets presented in this study can be found in online repositories. The names of the repository/repositories and accession number(s) can be found in the article/**Supplementary Material**.

## Supplementary material

Supplementary Data File 1 List of the 282 strains investigated in the study, with their clinical sources and MICs for various antibiotics.

Supplementary Data File 2 Genotypic features in 96 strains, with AMR genes, STs, and KO types in strains grouped on the basis of their AMR phenotype and CFDC susceptibility.

Supplementary Data File 3 OS mutations detected in 96 strains by WGS.
