# Supplementary material for: Genomic analysis reveals multi-level resistance network linking oxidative stress pathway mutations to cefiderocol resistance and clonal evolution in Klebsiella pneumoniae from the United Arab Emirates
Source: Front Cell Infect Microbiol. 2026 May 15;16:1820198. doi: 10.3389/fcimb.2026.1820198 (PMC13219037; doi:10.3389/fcimb.2026.1820198)
Supplement: Supplementary file 4 [file SupplementaryFile1.pdf]

msrB

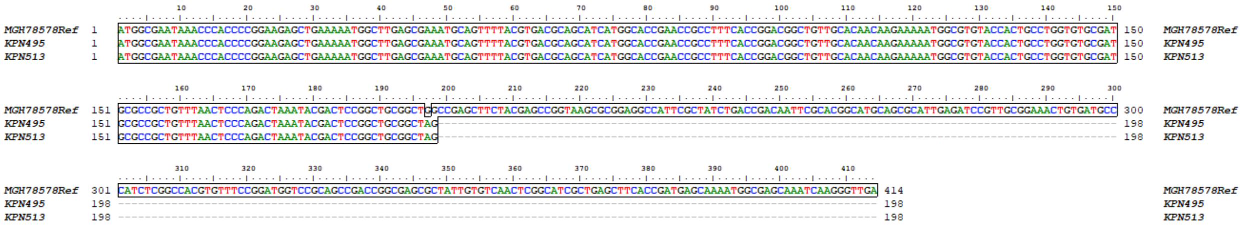

rpoS

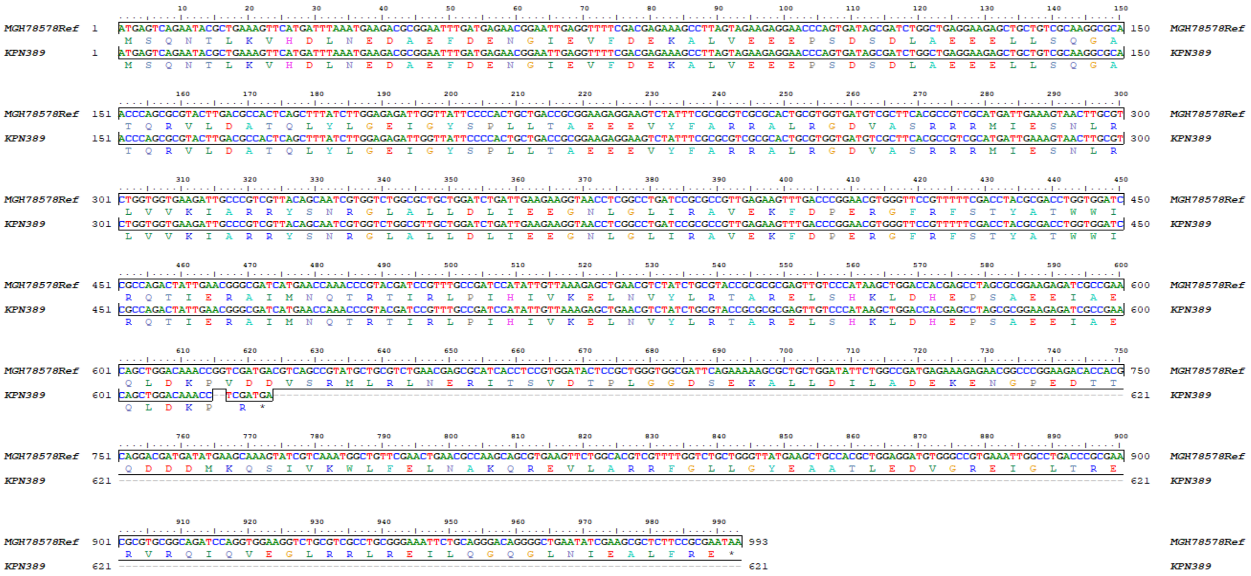

DNA repair genes with frameshift mutations leading to early stop codon.
